# E2F1 Maintains Gastric Cancer Stemness Properties by Regulating Stemness-Associated Genes

**DOI:** 10.1155/2021/6611327

**Published:** 2021-04-23

**Authors:** Yan Fu, Cheng'en Hu, Peizhun Du, Guangjian Huang

**Affiliations:** Department of General Surgery, Huashan Hospital, Fudan University, Shanghai 200040, China

## Abstract

**Purpose:**

To determine the regulatory role of E2F1 in maintaining gastric cancer stemness properties and the clinical significance of E2F1 in gastric cancer.

**Materials and Methods:**

We conducted a tumor spheroid formation assay to enrich gastric cancer stem-like cells. The protein and mRNA expression levels of genes were measured using Western Blot and qRT-PCR. Lentivirus-mediated overexpression and downregulation of E2F1 were performed to evaluate the effect of E2F1 on the stemness properties of gastric cancer cells. The effect of E2F1 on gastric cancer cell sensitivity of 5-Fu was evaluated using cell viability assay and TdT-mediated dUTP Nick-End Labeling staining. We also analyzed the association between E2F1 expression and clinical characteristics in gastric cancer patients. The KM plotter database was used to analyze the relationship between E2F1 and overall survival in GC patients.

**Results:**

We found that E2F1 expression was significantly higher in gastric cancer tissues than in the paired adjacent normal tissues (*p* < 0.05) and was positively correlated with tumor size (*p* < 0.05), T stage (*p* < 0.05), and differentiation degree (*p* < 0.05). KM plotter database demonstrated a close association between higher E2F1 expression level and worse overall survival of gastric cancer patients (*p* < 0.05). In vitro assay illustrated that E2F1 could regulate the expression of stemness-associated genes, such as BMI1, OCT4, Nanog, and CD44, and maintain the tumor spheroid formation ability of gastric cancer cells. E2F1 enhanced 5-Fu resistance in gastric cancer cells, and the E2F1 expression level was correlated with the prognosis of gastric cancer patients receiving 5-Fu therapy. The expression levels of stemness-associated genes were also significantly higher in gastric cancer tissues than the paired adjacent normal tissues (*p* < 0.05). A positive correlation was observed between E2F1 and BMI1 (*r* = 0.422, *p* < 0.05), CD44 (*r* = 0.634, *p* < 0.05), OCT4 (*r* = 0.456, *p* < 0.05), and Nanog (*r* = 0.337, *p* < 0.05) in gastric cancer tissues. The co-overexpression of E2F1 and stemness-associated genes was associated with worse overall survival.

**Conclusion:**

E2F1 plays a significant role in gastric cancer progression by maintaining gastric cancer stemness properties through the regulation of stemness-associated genes. The close association between E2F1 and poor prognosis of patients suggests that E2F1 could serve as a prognostic biomarker and a therapeutic target in gastric cancer patients.

## 1. Introduction

Gastric cancer (GC) is one of the most common gastrointestinal malignancies and the third leading cause of cancer-related deaths worldwide [1]. Despite the multidisciplinary therapeutic strategies in GC treatment, the prognosis of GC patients remains poor, with a 5-year overall survival (OS) rate of approximately 25% [2]. The prognosis of GC patients remains poor, largely due to drug resistance, frequent tumor relapse, and distant metastasis. Recent studies have suggested that a small group of cells possess stemness properties in cancer and are referred to as cancer stem-like cells (CSCs). CSCs are thought to have chemotherapy resistance, cause tumor metastasis and relapse, and might thus be a promising therapeutic target to overcome multidrug resistance and posttreatment relapse of cancer [3]. Takaishi et al. reported the use of CD44 as a surface marker for enriching GC stem-like cells (GCSCs) [4]. Stemness-associated genes, such as BMI1, Nanog, and OCT4, significantly contribute to the maintenance of stemness properties in GCSCs [5].

E2F family is a group of transcription factors playing significant roles in regulating the cell cycle. The family can be divided into two subfamilies based on their function: transcription repressors (E2F3b, E2F4, E2F5, E2F6, E2F7, and E2F8) and activators (E2F1, E2F2, and E2F3a) [6]. E2F1, a vital member of the E2F family, was originally recognized as an important cell cycle-regulating transcription factor [7]. Retinoblastoma family proteins (pRB) and dimerization proteins (DP), the two main E2F1 binding partners, affect the activity of E2F1 [8]. The transcriptional activation domain of the E2F1-DP complex can be blocked by E2F1-pRB (nonphosphorylated) interaction, preventing transcriptional activation of downstream target genes [9]. During the cell cycle progression, nonphosphorylated pRB, which binds with E2F1, can be phosphorylated by cyclin-dependent kinases (CDKs) to release E2F1, further transcriptionally activating genes involved in DNA synthesis, S-phase entry, and mitosis [9]. Besides, E2F1 also plays a critical role in regulating cell apoptosis [10], senescence [11], DNA damage [12], and metabolism [13]. Recently, Sun et al. reported the prognostic value of E2F in breast cancer [14]. However, the prognostic value of E2F1 in GC and its correlation with GC stemness properties remains to be demonstrated. In the current study, E2F1, which was significantly overexpressed in GC tissues, was negatively related to the prognosis of GC patients and contributed to GC progression by regulating the stemness properties of GC cells. This study indicates that E2F1 might serve as a promising prognostic biomarker and therapeutic target for GC.

## 2. Materials and Methods

### 2.1. Participants

A total of 62 paired GC tissues and conjugated tumor-adjacent tissues were obtained from Huashan Hospital between March 2016 and March 2017. Pathological diagnosis of GC was independently conducted by two pathologists. After resection, all tissue samples were snap-frozen in liquid nitrogen, followed by storage at −80°C. None of the enrolled patients had received radiotherapy or chemotherapy before surgery. Informed consent was obtained from each of the enrolled patients, and study protocols were approved by the Human Research Ethics Committee of Huashan Hospital. There were 11 female and 51 male patients, with an age range between 30 and 87 years and a median age of 63 years. A total of 16 cases showed a high and moderate degree of differentiation, while 46 cases showed a poor degree of differentiation. There were 19 cases without lymph node metastasis and 43 cases with lymph node metastasis. There were 18 cases in T1-2 and 44 cases in T3-4. Ki67 was less than 30% in 24 cases and more than 30% in 38 cases.

### 2.2. Data Collection

Baseline information of all patients, including pathological and clinical characteristics, surgery time, relapse information within two years after surgery, age, gender, tumor size, T stage, TNM stage, pathological grade, and positive lymph nodes, was collected. All experiments were repeated three times.

### 2.3. Cell Culture

The SGC-7901 and MKN-45 cell lines obtained from the Cell Bank of the Chinese Academy of Science (Shanghai, China) were cultured in DMEM medium (Gibco) supplemented with 10% fetal bovine serum, 100 IU/ml penicillin, and 100 *μ*g/mL streptomycin at 37°C in 5% CO_2_ atmosphere.

### 2.4. Cell Treatment

Cells at the logarithmic growth phase were harvested and seeded in a 6-well plate. Transfection was carried out when the cells reached 70% confluence according to the lipofectamine 3000 (Invitrogen, USA) instructions. The E2F1 silencing sequence was “CCGGCGCTATGAGACCTCACTGAATCTCGAGATTCAGTGAGGTCTCATAGCGTTTTTG”, which was loaded into the PLKO plasmid. The E2F1 CDS sequence was loaded into the PCDH plasmid for E2F1 overexpression in GC cells.

### 2.5. Cell Viability Assay

Cell viability was detected using CCK8 test kits following the manufacturer's instructions. After exposure to 5-Fu treatment for 3 days, cancer cells were incubated with a medium containing 10% CCK8 for 2 hours, and the absorbance was measured at 450 nm.

### 2.6. TdT-Mediated dUTP Nick-End Labeling (TUNEL) Assay

TUNEL assay was carried out to detect 5-Fu-induced apoptosis according to the manufacturer's instructions (Beyotime Biotechnology, China). Cyanine 3-labeled TUNEL-positive cells were imaged with a fluorescent microscope.

### 2.7. RNA Extraction and qRT-PCR

Total RNA of cultured cell lines and tissue samples was extracted using the Trizol reagent. PrimeScript RT Reagent Kit (TaKaRa) was used to generate the first-strand cDNA, and SYBR Premix Ex Taq (TaKaRa) was used to detect the mRNA expression level according to the manufacturer's instructions.  Table 1 summarizes the primers used for qRT-PCR.

### 2.8. Western Blot Assay

Cell protein samples were lysed in radioimmunoprecipitation assay (RIPA) buffer. The protein concentration was determined using the BCA kit. A loading buffer was added to the proteins and boiled at 100°C for 15 minutes. Polyacrylamide gel electrophoresis and membrane transfer were carried out as previously described [15]. The membrane was incubated with primary antibodies, OCT4 (Santa Cruze, USA), Nanog (Santa Cruze, USA), BMI1 (Santa Cruze, USA), CD44 (Abcam, USA), and GAPDH (Abcam, USA) overnight at 4°C. After incubation with secondary antibody for one hour at room temperature, enhanced chemiluminescence (ECL) reagent was used to visualize the protein bands using a Gel Doc EZ Imager. GAPDH was used as an internal reference.

### 2.9. Cell Migration and Invasion Assay

Cells at the logarithmic growth phase were harvested and seeded in transwell chambers with a serum-free medium to detect the migration ability. For invasion assay, Matrigel was added to the chambers, and the cells were seeded. After 48 h, the cells were fixed with 4% paraformaldehyde and stained with crystal violet. The invading and migrating cells were counted under a light microscope.

### 2.10. Immunofluorescence Staining

The cells were fixed in 4% freshly prepared paraformaldehyde for 15 minutes and treated with Triton X-100. Next, they were blocked with 10% bovine serum albumin (BSA) for 1 hour at room temperature. The cells were incubated with CD44 antibody (1 : 50) in 1% BSA overnight at 4°C, washed with TBS, and incubated with appropriate secondary antibody at room temperature for 1 hour in the dark. The cells were washed thrice and counterstained with 4′, 6-diamidino-2-phenylindole (DAPI) for 10 minutes. The cells were again washed three times and observed under an inverted fluorescent microscope.

### 2.11. Tumor Sphere Formation Assay

Cells were seeded in an ultralow adherent 96-well plate and cultured in serum-free DMEM/F12 medium containing B27 (1X), N2 (1X), epidermal growth factor (EGF) (20 ng/ml), and basic fibroblast growth factor (bFGF) (20 ng/ml).

### 2.12. The Prognostic Value of E2F1

The prognostic value of E2F1 for OS in GC was evaluated using the KM plotter (http://kmplot.com/analysis) database. We input the E2F1 sequences to obtain Kaplan–Meier survival plots. The hazard ratio (HR), 95% confidence interval (CI), and log-rank P were displayed on the web page. *p* < 0.05 was considered statistically significant.

### 2.13. Gene Expression Profiling Interactive Analysis (GEPIA) of E2F1 Expression in GC

The different expression levels of E2F1 between GC tissues and the adjacent normal tissues were analyzed using the GEPIA database (http://gepia.cancer-pku.cn/).

### 2.14. Data Analysis

SPSS 21.0 statistical software was used to analyze the data, which was presented as mean±SEM. A *t*-test was used to compare the expression levels of E2F1, OCT4, BMI1, NANOG, and CD44 in tumor tissues and paired adjacent normal tissues. Spearman's test was used to determine the correlation between the expression levels of E2F1 and stemness-associated genes. The single-factor analysis was carried out using *χ* test to analyze the factors affecting tumor relapse (a time point of two years after surgery). Factors with a *p* value <0.1 were included in multivariate logistic regression analysis. *p* value <0.05 was considered to be statistically significant.

## 3. Results

### 3.1. E2F1 Is Significantly Overexpressed in GC Tissues

**Figure 1:**
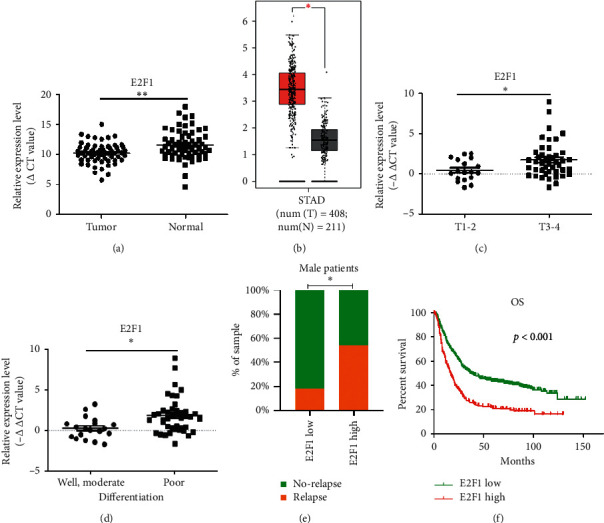
Increased expression of E2F1 in gastric cancer. (a) Increased E2F1 expression in gastric cancer tissue compared with normal stomach tissue. (b) GEPIA database demonstrates an increased expression level of E2F1 in gastric cancer tissues. (c) E2F1 mRNA expression level is higher in T3-4 stage patients compared with T1-2 stage patients. (d) E2F1 mRNA expression level is higher in poorly or moderately differentiated patients compared with well-differentiated patients. (e) Male patients with the higher expression level of E2F1 have a higher rate of tumor recurrence at a time point of two years after surgery. (f) Relationship between E2F1 expression and OS in gastric cancer patients.

The expression level of E2F1 was found to be significantly higher in GC tissues compared with that in adjacent stomach tissues (*p* < 0.05) (Figure 1(a)). The data in the GEPIA database also revealed increased expression of E2F1 in GC tissues (Figure 1(b)). Compared with T1-2 phase GC patients, T3-4 phase GC patients exhibited a higher expression level of E2F1 (*p* < 0.05) (Figure 1(c)). Besides, patients with poorly differentiated GC exhibited enhanced expression of E2F1 compared with patients with well-differentiated GC (*p* < 0.05) (Figure 1(d)), suggesting the potential role of E2F1 in GC progression. The association between E2F1 expression level and the risk of tumor relapse two years after surgery was also analyzed. GC patients with a higher E2F1 expression level exhibited a higher recurrence rate compared with those with lower E2F1 expression levels, even though the difference was not statistically significant (*p*=0.067) (Table 2). However, higher expression levels of E2F1 were found to be highly correlated with higher recurrence rates in male GC patients (Figure 1(e)). Further, we used the KM plotter database to analyze the correlation between E2F1 expression level and OS in GC patients, and the results revealed that patients with higher expression levels of E2F1 had a worse OS (HR = 1.91, *p* < 0.05) (Figure 1(e)). These findings revealed that increased expression of E2F1 in GC contributed to cancer progression and poor prognosis in GC patients.

### 3.2. Correlation between E2F1 mRNA Expression Level and the Clinical and Pathological Features of GC Patients

We further analyzed the relationship between E2F1 mRNA expression level and the clinical characteristics of patients, including gender, age, differentiation degree, lymph node metastasis, tumor size, T stage, and Ki67 positive rate. The results revealed significant correlations between E2F1 expression level and differentiation degree (*p* < 0.05), Tumor size (*p* < 0.05), and T stage (*p* < 0.05) (Table 3). Although the *p* value was greater than 0.05, patients with a higher expression level of E2F1 exhibited increased lymph node metastasis and Ki67 positive rate (Table 3). These results provide insights into the potential role of E2F1 in gastric cancer progression.

### 3.3. Factors Affecting the Risk of Tumor Recurrence

To analyze the factors affecting the risk of tumor recurrence after surgery, we performed a univariate analysis. The result suggested that T stage (*p* < 0.05) and Ki67 positive rate were both positively correlated with the risk of tumor recurrence within two years after surgery (*p* < 0.05) (Table 2). E2F1 was found to be a risk factor for tumor relapse in all GC patients (both male and female), even though the difference was not significant (*p*=0.067). However, a higher E2F1 expression level was correlated with a higher risk of tumor recurrence in male GC patients (Table 4), suggesting that E2F1 can serve as a predictive biomarker for tumor recurrence risk in male GC patients. This difference may be attributed to the relatively small number of female patients enrolled in this study. Therefore, studies with larger patient samples may be needed to further confirm the potential value of E2F1 in predicting the risk of tumor recurrence in GC patients. Furthermore, all factors with *p* < 0.1 in univariate analysis were enrolled into multivariate logistic regression analysis. The result revealed that the Ki67 positive rate was an independent predictive factor for tumor relapse within two years after surgery (Table 5).

### 3.4. Tumor Spheroid Cells Exhibited Increased Expression of Stemness-Associated Genes

**Figure 2:**
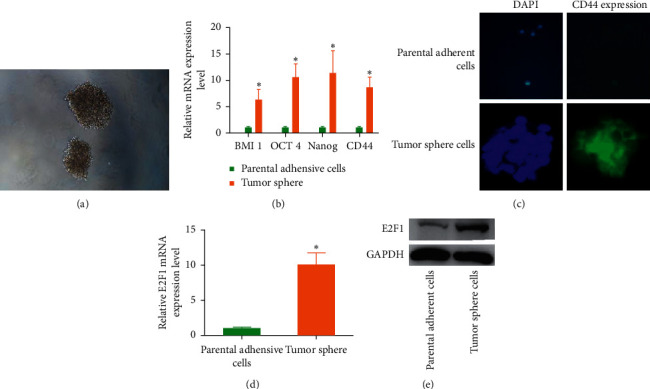
Enrichment of gastric cancer stem-like cells. (a) Tumor serum-free culture. (b) The mRNA expression level of stemness-associated genes in parental and tumor sphere cells. (c) Immunofluorescence staining of CD44 in parental adherent cells and tumor sphere cells. (d) The mRNA expression level of E2F1 in parental and tumor sphere cells. (e) The protein expression level of E2F1 in parental and tumor sphere cells.

GCSCs can be enriched using tumor sphere formation assay [5] (Figure 2(a)). Compared with parental cells, tumor spheroid cells exhibited increased expression of stemness-associated genes, such as BMI1, OCT4, Nanog, and CD44 (Figure 2(b)). CD44 is regarded as a surface marker for GCSCs. Compared with parental adherent cells, tumor spheroid cells showed increased expression of CD44 (Figure 2(c)), suggesting the stemness properties of tumor sphere cells. We also found that E2F1 expression level was also significantly increased in GCSCs (Figures 2(d) and 2(e)), indicating the potential role of E2F1 in regulating GC stemness properties.

### 3.5. E2F1 Contributes to Stemness Properties in GC

**Figure 3:**
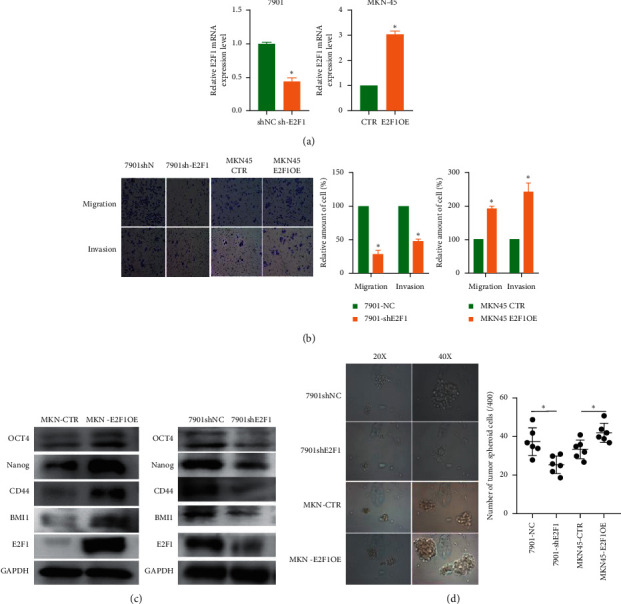
E2F1 contributes to stemness-property maintaining through regulating stemness-associated genes. (a) Downregulation of E2F1 in 7901 cells and overexpression of E2F1 in MKN-45 cells. (b) E2F1 increases migration and invasion ability in gastric cancer. (c) E2F1 regulates stemness-associated genes, such as OCT4, Nanog, BMI1, and CD44. (d) E2F1 regulates tumor sphere formation ability of gastric cancer.

To further determine the regulatory role of E2F1 in maintaining GC stemness properties, E2F1 expression was overexpressed in the MKN45 cell line and downregulated in the 7901 cell line (Figure 3(a)). E2F1 knockdown inhibited the migration and invasion ability of 7901 cells, while E2F1 overexpression improved the migration and invasion ability of MKN45 cells (Figure 3(b)). Western Blot analysis revealed that E2F1 was a regulator of stemness-associated genes in GC, such as OCT4, Nanog, BMI1, and CD44 (Figure 3(c)). Overexpression of E2F1 confers increased tumor sphere formation ability in MKN45 cell line, while E2F1 knockdown inhibits tumor sphere formation ability in 7901 cell line (Figure 3(d)). These results indicated that E2F1 maintains stemness by regulating stemness-associated genes in GC.

### 3.6. E2F1 Regulate 5-Fu Sensitivity in GC

**Figure 4:**
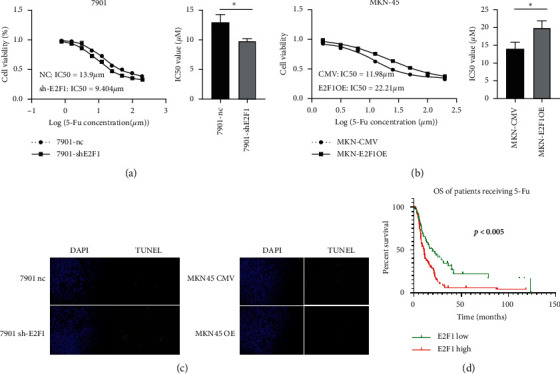
E2F1 regulates 5-Fu sensitivity in GC. (a) Downregulation of E2F1 in 7901 sensitized GC cells to 5-Fu. (b) Upregulation of E2F1 reduced the sensitivity of GC cells to 5-Fu. (c) Downregulation of E2F1 increased 5-Fu-induced apoptosis in GC cells whereas upregulation of E2F1 decreased 5-Fu-induced apoptosis in GC cells. (d) Compared with patients with high expression of E2F1, patients with low expression of E2F1 have a better OS after receiving 5-Fu chemotherapy.

Drug resistance is a typical feature of CSC; therefore, we detected the effect of E2F1 on GC sensitivity to 5-Fu, a commonly used chemotherapeutic drug for GC treatment. Our results revealed that knockdown of E2F1 significantly decreased the IC50 of GC cells (Figure 4(a)), which significantly increased when E2F1 was overexpressed (Figure 4(b)). TUNEL assay also revealed that E2F1 silencing augmented the apoptosis induced by 5-Fu, whereas E2F1 overexpression attenuated the apoptosis induced by 5-Fu in GC cells (Figure 4(c)). Using the KM plotter database, we also found that a higher expression level of E2F1 was correlated with a worse OS in patients receiving 5-Fu-based chemotherapy (Figure 4(d)). These results suggested that E2F1 contributes to 5-Fu resistance in GC and has the potential to serve as an indicator to predict 5-Fu sensitivity in GC patients.

### 3.7. Correlation of E2F1 Expression with Stemness-Associated Genes in GC

**Figure 5:**
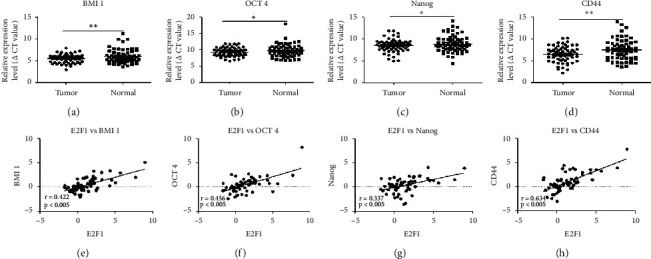
Expression of stemness-associated gens in gastric cancer, and their correlation with E2F1. (a) BMI1, (b) OCT4, (c) Nanog, and (d) CD44 expression in gastric cancer tissue and grouped normal tissue. Correlation between E2F1 expression and stemness-associated genes (e) E2F1 versus BMI1, (f) E2F1 versus OCT4, (g) E2F1 versus Nanog, and (h) E2F1 versus CD44.

Expression of stemness-associated genes in GC tissues, such as BMI1, OCT4, Nanog, and CD44, was detected using qRT-PCR. Compared with adjacent normal tissues, GC tissues had significantly higher expression levels of BMI1 (*p* < 0.05), OCT4 (*p* < 0.05), Nanog (*p* < 0.05), and CD44 (*p* < 0.05) (Figures 5(a)–5(d)). A positive correlation between E2F1 and BMI1 (*r* = 0.422, *p* < 0.005), OCT4 (*r* = 0.456, *p* < 0.005), Nanog (*r* = 0.337, *p* < 0.005), and CD44 (*r* = 0.643, *p* < 0.005) was observed (Figures 5(e)–5(h)), suggesting that E2F1 might be associated with stemness properties in GC.

### 3.8. Correlation of Stemness-Associated Gene Expression with OS in Different GC Patients with E2F1 Expression

**Figure 6:**
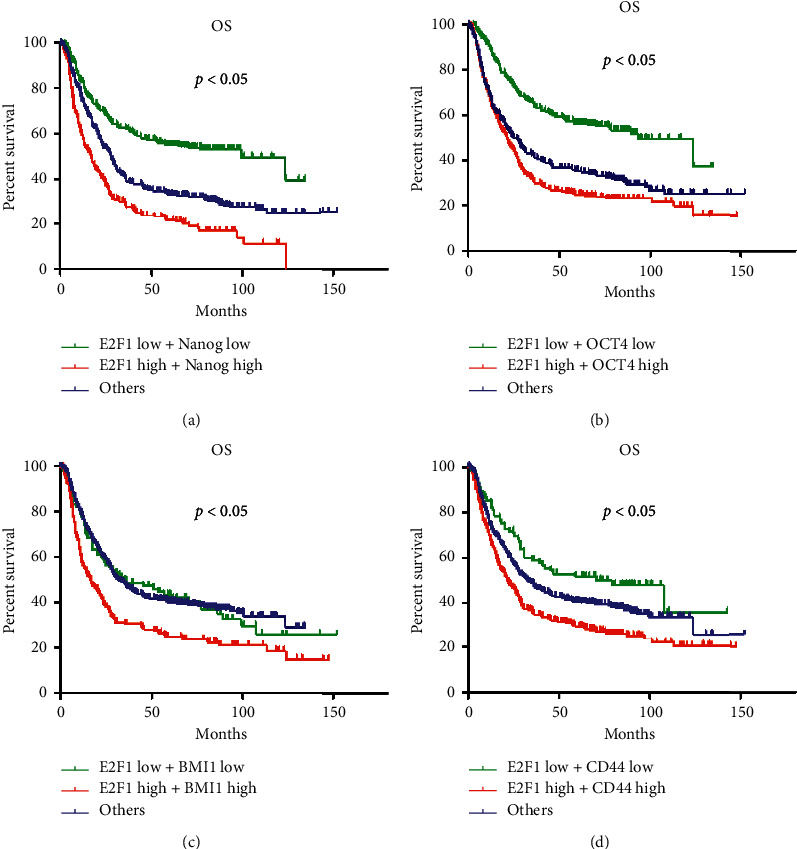
Kaplan–Meier analysis of GC patients with different expression levels of E2F1 and stemness-associated genes. All patients were divided into three groups according to the expression level of (a) E2F1 and Nanog, (b) E2F1 and OCT4, (c) E2F1 and BMI1, and (d) E2F1 and CD44. The high group referred to patients with high expression of both E2F1 and stemness-associated gene; the low group referred to patients with low expression of both E2F1 and stemness-associated gene; the middle group referred to the remaining patients. OS differences among the three groups were compared using Kaplan–Meier analysis.

Patients were divided into three groups according to the expression level of E2F1 and Nanog (Figure 6(a)), E2F1 and OCT4 (Figure 6(b)), E2F1 and BMI1 (Figure 6(c)), and E2F1 and CD44 (Figure 6(d)). The high group referred to patients with high expression of both E2F1 and stemness-associated genes; the low group referred to patients with low expression of both E2F1 and stemness-associated genes, and the middle group referred to the rest of the patients. We found that patients with low expression of both E2F1 and Nanog had better OS compared with co-overexpression of E2F1 and Nanog group (Figure 5(a)). Similar results were also observed in the three other groups (Figures 5(b) and 5(d)), suggesting that co-overexpression of E2F1 and stemness-associated genes serves as prognostic markers for GC patients. Compared with E2F1 alone, co-overexpression of E2F1 and Nanog or OCT4 was a better prognostic indicator for GC patients.

## 4. Discussion

As one of the most investigated E2F families in cancer, E2F1 was originally recognized as an important regulator of the cell cycle [16]. E2F1 is reported to interact with histone acetyltransferase (HAT) adaptor protein; TRRAP recruits them to chromatin to promote hyperacetylation of H3 and H4, further facilitating the expression of its downstream genes [17]. Increasing evidence suggests the multifunction role of E2F1 in addition to cell cycle regulation. E2F1 contributes to DNA repair by associating with TOPBP1 to recruit the BRG1/BRM components of the SWI/SNF to its downstream genes, thus preventing the apoptotic signaling pathway [18]. However, another study suggested that E2F1 could induce the apoptosis of Saos-2 cells undergoing DNA damage through several mechanisms, such as inducing inactivation of antiapoptotic signals and activating the death receptor pathway and intrinsic apoptotic pathway [19]. Other studies report that E2F1 also plays an essential role in metabolic reprogramming in cancer cells, causing repressed glucose oxidation in mitochondria and enhanced glycolysis [20]. These studies indicate the crucial role E2F1 plays in mediating multiple hallmarks of cancer.

In human tumors, the enhancement of E2F1 activity can be attributed to gene amplification, overexpression, posttranslational modification, and release from RB [21]. Aberrant activation of E2F1 promotes tumor progression through cell cycle-stimulating effect-mediated unrestrained cell proliferation, leading to the unfavorable prognosis of cancer patients [21, 22]. Recent studies also indicate that E2F1 is significantly upregulated in late-stage tumors and contributes to tumor invasion and migration [23, 24]. Transgenic mice overexpressing E2F1 were found to be more susceptible to tumorigenesis due to the loss of normal regulation of the cell cycle [25], further suggesting the oncogenic role of E2F1 in cancer. However, several studies report conflicting results that challenge the claim that E2F1 plays an oncogenic role [26]. For example, E2F1 is regarded as a checkpoint regulatory protein, involved in DNA damage stress response, and plays an important role in promoting apoptosis [12]. Yamasaki et al. reported that E2F1 knockout-transgenic mice exhibited decreased levels of apoptosis and a tendency to spontaneously produce tumors, which was associated with decreased expression of downstream apoptosis-related genes following the inactivation of E2F1 [27]. In liver cancer, a significant positive correlation between the expression level of E2F1 and the level of tumor apoptosis has been reported, suggesting that E2F1 can play a tumor-suppressive role through proapoptosis [28]. Therefore, E2F1 plays a conflicting role in cancer, which is highly dependent on the cellular context.

In GC, E2F1 plays a significant role in maintaining malignant phenotypes. Previously, two studies suggested that increased expression of E2F1 inhibited proliferation and induced the apoptotic response of GC [29, 30], suggesting an inhibitory role of E2F1 in GC progression. However, recent evidence indicates the oncogenic role of E2F1 in GC. For example, E2F1 knockdown enhances drug sensitivity of 7091 cell line to cisplatin and doxorubicin, an indication that increased expression of E2F1 is associated with multidrug resistance in GC [31]. siRNA-mediated inhibition of E2F1 significantly suppresses the proliferation and invasion ability of MGC-803 cells, suggesting a stimulative effect of E2F1 in GC progression [32]. Besides, recent studies have identified the oncogenic role of E2F1 in gastric cancer [33–35]. E2F1 is reported to have dispositional properties that only appear under satisfying conditions with regard to cellular and molecular status (feedback signals, interaction with cofactors, and subcellular localization) [24]. In the present study, we found that the E2F1 expression level was significantly higher in GC tissues compared with paired adjacent normal tissues. Besides, the E2F1 expression level was positively correlated with poor differentiation, tumor size, and T stage. E2F1 serves as a predictor of tumor recurrence risk in male GC patients within two years after surgery. Kaplan–Meier curves showed that GC patients with the higher expression level of E2F1 had a worse OS. All these findings suggest that E2F1 is involved in GC progression and is a potential predictor for the poor prognosis of GC patients.

Recently, CSCs have gained increasing attention for their contribution to tumor-initiating, chemotherapy resistance, metastasis, and relapse [36]. In GC, GCSCs are closely associated with the poor prognosis of GC patients. CD44+ circulating tumor cells (CTCs) may act as a prognosis predictor in GC due to their close association with tumor metastasis and relapse [37]. The expression level of CD44, a surface marker for GCSCs, is positively associated with TNM grade, remote metastasis, and relapse risk in GC [38]. GCSCs rely on several stemness-associated genes to maintain their stemness properties, such as BMI1, OCT4, and Nanog. In the present study, we found that E2F1 contributed to GC stemness properties by regulating stemness-associated genes, such as BMI1, OCT4, Nanog, and CD44. Drug resistance is a typical feature of CSC. Our results revealed that E2F1 also contributed to 5-Fu resistance in GC, and E2F1 was a potential predictor of 5-Fu treatment sensitivity in GC patients. Previous studies have reported the direct role of E2F1 in regulating stemness-associated genes in several other tumors. For example, Lu et al. reported that E2F1 could bind to the promoter region of Nanog to initiate its transcription in breast cancer [39]. Using chromatin immunoprecipitation (ChIP) assay and luciferase gene reporter assay, they confirmed that E2F1 endogenous binds with the region sites (−586∼−576) of Nanog promoter to initiate its transcription. Besides, E2F1 could bind to the promoter of BMI1 to initiate its transcription in neuroblastomas [40]. Therefore, there is a possibility that E2F1 directly activates the transcription of stemness-associated genes in GC. In GC tissues, E2F1 was positively correlated with BMI1 (*r* = 0.422, *p* < 0.005), OCT4 (*r* = 0.456, *p* < 0.005), Nanog (*r* = 0.337, *p* < 0.005), and CD44 (*r* = 0.643, *p* < 0.005), indicating a relationship between E2F1 and stemness properties in GC.

In conclusion, E2F1 is closely associated with the prognosis of GC patients and exhibits the potential to serve as a prognostic biomarker for GC patients. The oncogenic effect of E2F1 in GC may be partially attributed to their regulatory role in maintaining stemness properties by regulating stemness-associated genes, thus, suggesting that E2F1 might serve as a target for developing GCSC-targeted anticancer therapy.

## Figures and Tables

**Table 1:** The primer sequences of real-time PCR.

Gene	Primer sequences (5′-3′)
E2F1	Forward: CATCCCAGGAGGTCACTTCTG
	Reverse: GACAACAGCGGTTCTTGCTC
OCT-4	Forward: CAGTGCCCGAAAGCCACAC
	Reverse: GGAGACCCAGCAGCGTCAAA
Nanog	Forward: CCCAAAGGCAAACAACCCACTTCT
	Reverse: AGCTGGGTGGAAGAGAACACAGTT
BMI1	Forward: CGCTTGGCTCGCATTCATTT
	Reverse: AAAAATCCCGGAAAGAGCAGC
CD44	Forward: TCCATCAAAGGCATTGGGCAG
	Reverse: AACCTGCCGCTTTGCAGGTGT
GAPDH	Forward: GAGTAAGACCCCTGGACCAC
	Reverse: AACTGGTTGAGCACAGGGTA

**Table 2:** Univariate analysis of the characteristic of GC patients for tumor relapse risks (^∗^*∗p* < 0.05).

Factor		No relapse	Relapse	*χ* value	*p* value
E2F1	High	15	13	3.595	0.067
	Low	26	8		
Sex	Male	33	18	0.26	0.735
	Female	8	3		
Age	≥63	21	15	2.329	0.176
	<63	20	6		
Tumor size (cm)	≥4	22	15	1.822	0.274
	<4	19	6		
Differentiation	Poor	27	19	4.397	0.063
	Well, moderate	14	2		
Lymph node metastasis	+	25	18	3.999	0.079
	−	16	3		
T Stage	T3-4	24	20	9.079	0.003^∗^*∗*
	T1-2	17	1		
Ki67	>30%	19	19	10.828	0.001^∗^*∗*
	≤30%	21	2		

**Table 3:** The association between E2F1 mRNA expression and clinicopathological characteristics in gastric cancer (^∗^*∗p* < 0.05).

Clinicopathological characteristics	Number of patients	E2F1 (−△△CT value)	*p* value
Gender			0.3910
Male	51	1.460 + 0.2977	
Female	11	0.8645 + 0.5380	
Age (Years old)			0.9699
<63	26	1.366 + 0.3811	
≥63	36	1.346 + 0.3637	
Differentiation			0.0368^∗^*∗*
Well, moderate	16	0.4294 + 0.3463	
Poor	46	1.676 + 0.3214	
Lymph node metastasis			0.1303
0	19	0.7547 + 0.3995	
≥1	43	1.619 + 0.3300	
Tumor size			0.0125^∗^*∗*
<4	25	0.5664 + 0.2620	
≥4	37	1.887 + 0.3815	
T Stage			0.0241^∗^*∗*
T1-2	18	0.4361 + 0.3125	
T3-4	44	1.730 + 0.3330	
Ki67 positive rate (%)			0.0913
≤30	24	0.7958 + 0.4324	
>30	38	1.707 + 0.3218	

**Table 4:** Univariate analysis of the characteristic of GC patients for tumor relapse risks in male patients (^∗^*∗p* < 0.05).

Factor		No relapse	Relapse	*χ* value	*p* value
E2F1	High	11	13	7.07	0.01^∗^*∗*
	Low	22	5		
Age	≥63	18	14	2.689	0.135
	<63	15	4		
Tumor size (cm)	≥4	17	14	3.370	0.080
	<4	16	4		
Differentiation	Poor	19	16	5.304	0.028^∗^*∗*
	Well, moderate	14	2		
Lymph node metastasis	+	19	16	5.304	0.028^∗^*∗*
	−	14	2		
T Stage	T3-4	20	17	6.696	0.010^∗^*∗*
	T1-2	13	1		
Ki67	>30%	16	17	10.773	0.002^∗^*∗*
	≤30%	17	1		

**Table 5:** Multivariant logistic regression analysis of the characteristic of GC patients for tumor relapse risks (^∗^*∗p* < 0.05).

	*β*	S.E,	Wals	*p* value	OR	95% CI of OR	
E2F1 high expression	0.099	0.704	0.020	0.888	1.104	0.278	4.387
Poor differentiation	1.22.	0.973	1.571	0.210	3.386	0.503	22.813
Lymph node metastasis	0.283	0.939	0.091	0.763	1.327	0.210	8.365
T Stage 3-4	2.255	1.194	3.571	0.059	9.540	0.920	98.956
Ki67 ≥ 30%	2.225	0.876	6.451	0.011^∗^*∗*	9.255	1.662	51.541

## Data Availability

The data used to support the findings of this study are included within the article.
